# Jasmonates, Ethylene and Brassinosteroids Control Adventitious and Lateral Rooting as Stress Avoidance Responses to Heavy Metals and Metalloids

**DOI:** 10.3390/biom11010077

**Published:** 2021-01-08

**Authors:** Camilla Betti, Federica Della Rovere, Diego Piacentini, Laura Fattorini, Giuseppina Falasca, Maria Maddalena Altamura

**Affiliations:** 1 Department of Medicine, University of Perugia, Piazzale Menghini 8/9, 06132 Perugia, Italy; 2 Department of Environmental Biology, Sapienza University of Rome, Piazzale Aldo Moro 5, 00185 Rome, Italy; federica.dellarovere@uniroma1.it (F.D.R.); diego.piacentini@uniroma1.it (D.P.); laura.fattorini@uniroma1.it (L.F.); giuseppina.falasca@uniroma1.it (G.F.); mariamaddalena.altamura@uniroma1.it (M.M.A.)

**Keywords:** jasmonates, brassinosteroids, ethylene, auxin, nitric oxide, adventitious rooting, lateral rooting, cadmium and arsenic soil pollution

## Abstract

Developmental and environmental signaling networks often converge during plant growth in response to changing conditions. Stress-induced hormones, such as jasmonates (JAs), can influence growth by crosstalk with other signals like brassinosteroids (BRs) and ethylene (ET). Nevertheless, it is unclear how avoidance of an abiotic stress triggers local changes in development as a response. It is known that stress hormones like JAs/ET and BRs can regulate the division rate of cells from the first asymmetric cell divisions (ACDs) in meristems, suggesting that stem cell activation may take part in developmental changes as a stress-avoidance-induced response. The root system is a prime responder to stress conditions in soil. Together with the primary root and lateral roots (LRs), adventitious roots (ARs) are necessary for survival in numerous plant species. AR and LR formation is affected by soil pollution, causing substantial root architecture changes by either depressing or enhancing rooting as a stress avoidance/survival response. Here, a detailed overview of the crosstalk between JAs, ET, BRs, and the stress mediator nitric oxide (NO) in auxin-induced AR and LR formation, with/without cadmium and arsenic, is presented. Interactions essential in achieving a balance between growth and adaptation to Cd and As soil pollution to ensure survival are reviewed here in the model species *Arabidopsis* and rice.

## 1. Introduction

Developmental plasticity allows plants to colonize a wide range of different ecosystems by promoting adaptation through a convergence of developmental and environmental signaling networks. Plants are characterized by a post-embryonic mode of development, based on the continuous activity of stem cells either embedded in the meristems, or deriving by a dedifferentiation process of adult cells. Moreover, plant development is modular, with the same type of organs initiated repeatedly from the same stem cell system. As plants are sessile organisms, cell identity is generally determined by positional cues, but it can also change during development until terminal differentiation [1,2]. In changing environments, e.g., in the presence of soil pollution, the plant endogenous regulatory system needs to adapt the morphogenic/organogenic response to the available resources, or to the avoidance of the environmental risk, while conserving the overall functional patterns necessary for survival. The regulation of cell fate transition dynamics is a common mechanism both in animals and plants, despite more attention being given to the animal field so far ([1] and references therein). Interestingly, the development of the root system in polluted soil is an attractive model to reveal the plant regulatory scenario in response to the environment. This is because roots, which generally develop in the soil, are the first organs to get in contact with pollutants. Therefore, plant survival strictly depends on efficient root response and functionality. The root system is formed by the embryonic-in-origin primary root (PR), the post-embryonic lateral roots (LRs), and the adventitious roots (ARs). The LRs originate by anticlinal divisions in the pericycle founder cells of either the PR or the ARs. The ARs are generally post-embryonic in origin and come from aerial organs and the non-pericycle tissues of the PR [3]. The term ARs include roots formed at the root–shoot junction, crown roots formed by nodes below ground, brace roots from nodes above ground, stem internode roots, and roots arising from the hypocotyl [4]. In some crops, like wheat and rice, ARs may also be embryonic in origin and are called seminal roots [5,6]. When present in the root system, the ARs anchor the plant to the soil, and facilitate gas exchange and uptake of minerals, water and O_2_. The formation of ARs is also essential for vegetative propagation in planta and in vitro, is a determinant for breeding programs, and is crucial for plant survival in altered environments, including polluted soils. For example, AR formation is a common adaptive response in rice growing in flooded soils [7].

In in vitro formed ARs, tissue origin may be different depending on the explant type and plant species. AR development can be mediated or not by an intermediate phase of callus formation, having an indirect or direct origin, respectively. In both cases, auxin is the main inducer in this process [8,9].

**Figure 1:**
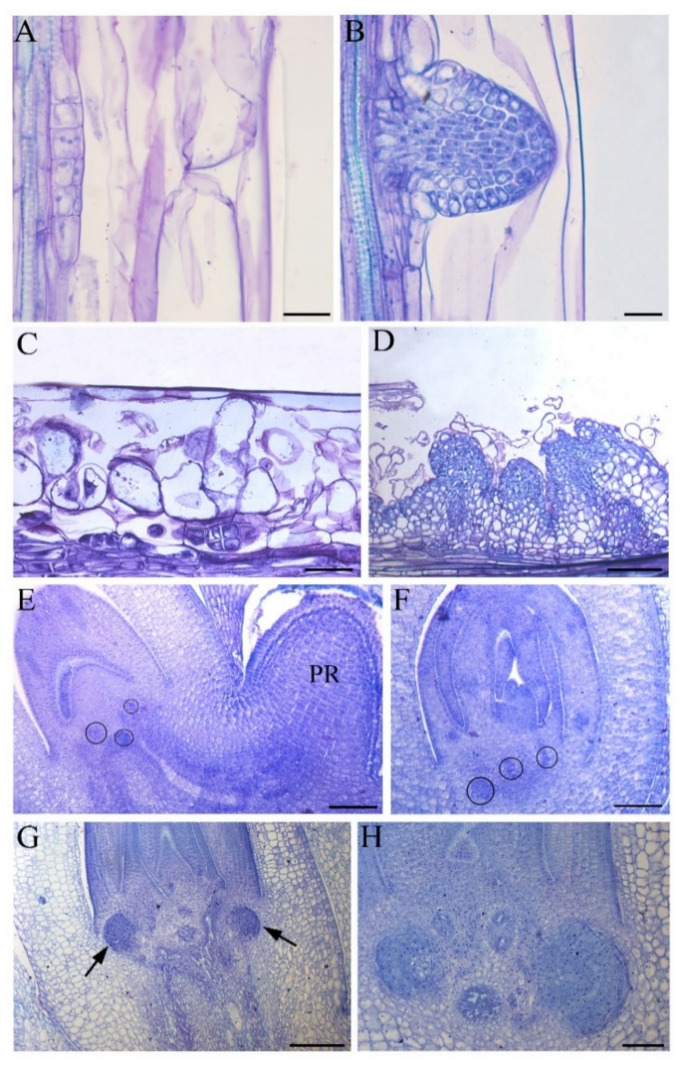
Adventitious root (AR) formation in hypocotyls (**A**,**B**) and thin cell layer explants (**C**,**D**) of *Arabidopsis*, and in the mature embryo of rice (**E**–**H**). First anticlinal cell divisions in the hypocotyl pericycle (**A**), and AR-primordium formation (**B**) in *Arabidopsis* seedlings. Radial longitudinal sections of *Arabidopsis* thin cell layers (TCLs) excised from the inflorescence stem and cultured under darkness in the presence of 10 μM of indole-3-butyric acid (IBA). Periclinal cell divisions in the stem endodermis and meristemoid formation from the most superficial derivatives at day 5 (**C**), and further development of these meristemoids into AR primordia ((**D**), day 10). Mature embryos of rice showing meristematic clumps (circles) initiating AR primordia at the scutellar node (**E**,**F**). Adventitious root primordia at the scutellar node at day 3 of germination ((**G**), arrows). Dome-shaped adventitious root primordium with cap differentiation ((**H**), day 4 of germination). PR, primary root. Longitudinal sections stained with toluidine blue. Bars = 20 µm (**A**,**B**), 50 µM (**C**), 100 µM (**H**) and 200 µm (**D**–**G**).

The model dicot plant *Arabidopsis thaliana* has a tap root system with a well-developed PR with LRs and no or just a poor number of ARs. The ARs are formed at the hypocotyl base from anticlinal divisions in pericycle cells (Figure 1A,B) [10]. However, other tissues of *Arabidopsis*, such as the stem endodermis, are also capable of forming ARs when cultured in vitro. This occurs in dark-grown thin cell layers (TCLs) longitudinally excised from the inflorescence stem and formed by the epidermis, the cortical parenchyma, and the endodermis. When treated with auxin, alone or combined with cytokinin, these explants form ARs without an intermediate phase of callus formation (Figure 1C,D [10,11,12]).

Differently from *Arabidopsis,* in the model plant *Oryza sativa*, different types of explants are able to form ARs only after callus formation. However, the transferability of the results obtained in *Arabidopsis* on de novo root formation to rice crop is highly promising [13].

Similarly to many other monocot crops, but again in contrast to *Arabidopsis*, *Oryza sativa* has a fibrous root system with a short-living PR and a high number of ARs and LRs derived from these ARs. The rice ARs are embryonic and post-embryonic in origin [5], despite their embryonic origin being questioned by some authors [14,15]. Our recent histological analyses on mature embryos show that two to three AR-meristematic clumps arise from the pro-vascular strand just above the scutellar node (Figure 1E,F). The AR-meristematic clumps differentiate the apical dome only after the radicle (i.e., the future PR) protrusion from the caryopsis (Figure 1G,H). The post-embryonic ARs are instead numerous, e.g., several hundred in field-grown plants ([15], and other references therein), and derive from the ground meristem adjacent to the peripheral stem vascular bundles [15]. Interestingly, the initial cells of the post-embryonic ARs originate from one or two periclinal divisions in the innermost ground meristem cells and form irregular-in-shape meristematic clumps; this is similar to what occurs during embryonic AR formation (Figure 1E,F). Gradually, each clump differentiates the various meristematic tissues of the AR-primordium, thus acquiring the fundamental dome-shaped structure only late in development [15].

In *Arabidopsis* and rice, the tissue initiating the ARs by asymmetric cell divisions (ACDs), i.e., divisions separating the fate of the derivative cells, is different, as are the orientations of the first cell division plane in the founder cells. Despite that, there are also similarities between the two species; firstly, recent studies have identified common key regulators in the auxin transcriptional networks involved in AR formation in vitro [13]. Furthermore, a similar involvement of auxin biosynthetic and transport genes in AR formation in planta, both under normal and metal/metalloid stress conditions, has been demonstrated [16,17]. Secondly, a high homology in the ACDs genetic control has been found, with ORYZA SATIVA SCARECROW 1 (OsSCR1) and ORYZA SATIVA SHORT-ROOT 1 (OsSHR1) rice transcription factors being functional equivalents of the *Arabidopsis* AtSCR and AtSHR [5]. Thirdly, the stress caused by the soil pollutants cadmium (Cd) and arsenic (As) similarly affects the quiescent center (QC) activity in the AR apical meristem [16,17].

Of note, the same abiotic stress can induce differences in the adventitious rooting process in the two model plants, even if the same hormones/transcription factors are involved. For example, the stress caused by flooding results into promotion of AR formation in rice and AR elongation in *Arabidopsis.* This different behavior depends on a flooding-induced trapping effect of ethylene (ET) in both species, occurring through the same APETALA2/ETHYLENE-RESPONSE FACTOR (AP2/ERF) transcription factors [18,19].

Recent advances in knowledge on lateral and adventitious rooting in *Arabidopsis* and rice are described here in response to the soil pollutants Cd and As. The two species were chosen because they are the most studied model plants from a molecular and genetic point of view, in dicots and monocots respectively. A comprehensive overview of the activity of several hormones interacting with auxin, i.e., jasmonates (JAs), ethylene (ET), and brassinosteroids (BRs), is provided. These hormones were selected because they are essential morphogenic signals, which cooperate with nitric oxide (NO) in the mechanisms that control the balance between growth and adaptation to soil pollution to ensure survival. Adventitious and lateral rooting were described in detail because they represent excellent examples of growth plasticity in response to Cd and As. The two pollutants were chosen because they are stress inducers commonly present in the environment in which rice is cultivated. The final aim is to provide inputs for transferring the broad scientific knowledge about *Arabidopsis* to rice, due to its huge agronomic value for humans and their health.

## 2. Cadmium and Arsenic Alter the Root System by Affecting Auxin Biosynthesis and Transport

Soil pollution can have adverse effects on the root system by either depressing lateral and/or adventitious rooting or enhancing their formation as a stress avoidance/survival response. In *Arabidopsis* and rice the root system is composed of a PR, some LRs and ARs. However, the root architecture is different in the two plants. A long-lived PR, short LRs and very few ARs form the root system in *Arabidopsis*, whereas in rice a short-lived PR and many embryonic and post-embryonic ARs, forming most of the LRs, are present. This different architecture can affect root system plasticity in response to soil pollution and can explain the different fitness and behavior of the two species in polluted environments depending on the root type involved in the response.

In *Arabidopsis,* the exposure to Cd, alone or combined with other toxic elements, such as Cu and Zn, alters the morphology and histological organization of the PR and changes the root architecture, thereby negatively affecting plant growth [20,21,22,23]. Moreover, Cd inhibits the PR by reducing the size of the root apical meristem due to a decrease in the quiescent center (QC) cell number [24,25]. By contrast, As causes PR elongation but alters peroxisome distribution in the apex, as Cd, albeit to a lesser extent [26].

The QC is the most sensitive site to Cd and/or As toxicity, also in ARs and LRs of *Arabidopsis*. In fact, both pollutants inhibit QC establishment and functioning and cause either an arrest of primordium outgrowth, or a precocious differentiation of the primary tissues [16]. However, the two pollutants affect AR and LR density in an opposite way, with Cd increasing and As reducing AR and LR density [16,27,28].

In rice, Cd induces anomalous divisions in the initial cells of LRs and no, or irregular, QC establishment, thus blocking the formation of LR primordia (LRPs) at the onset or their development. This also causes precocious differentiation of the aerenchyma in the few elongated LRs. Arsenic causes the formation of LRPs with a QC not properly organized, and the presence of differentiated cells even in the apical meristem. Contrariwise, the combined exposure to As and Cd induces diffuse plasmolysis in cortical cells and in the endodermis of ARs [17].

Altogether, these results highlight that in the root system of *Arabidopsis* and rice the cells most susceptible to Cd and As toxicity are those of the QC and the initials with ACDs [16,17].

Numerous phytohormones act in concert in regulating plant development and environmental responses. Auxin (indole-3-acetic acid, IAA) is a key phytohormone in PR, LR, and AR formation [10,29], and common mechanisms of auxin biosynthesis and polar transport in roots have been suggested for rice and *Arabidopsis* [30]. In *Arabidopsis*, AUXIN TRANSPORTER PROTEIN 1 (AUX1) and AUXIN TRANSPORTER-LIKE PROTEIN 3 (LAX3) are involved in IAA transport during LR and AR formation and development [10,31,32]. AUX1 is also involved in regulating plant responses to abiotic stresses [33]. Moreover, the transcription factors SHR and SCR are key regulators of PR, LR, and AR stem cell definition/maintenance, jointly regulating QC identity genes ([32] and references therein). The two transcription factors also cooperate with AUX1 in controlling ACDs leading to AR formation in the hypocotyl pericycle [32,34]. Very recently, the role of AUX1 has been further stressed because its expression has been revealed to be crucial for an adequate AR establishment also in pre-etiolated flooded seedlings [21].

**Figure 2:**
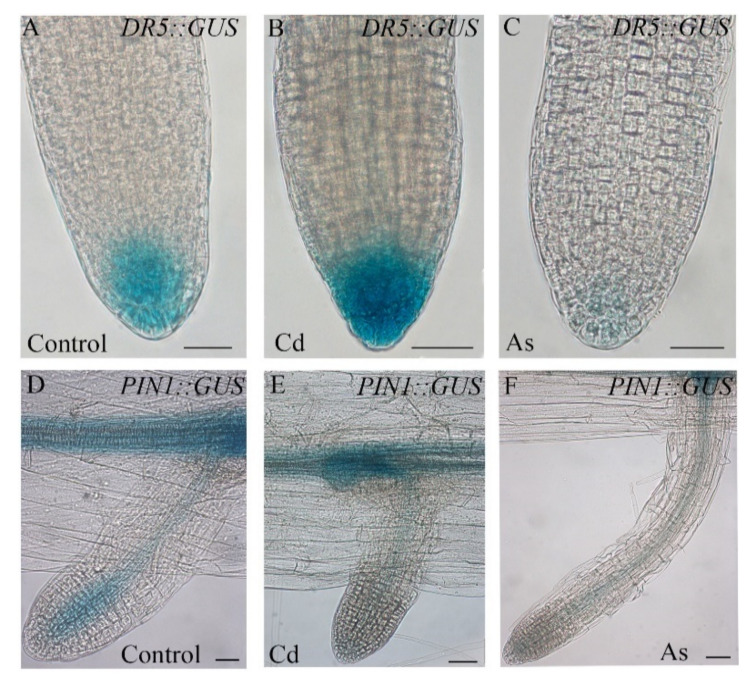
Expression patterns of *DR5::GUS* and *PIN1::GUS* in lateral roots (LRs) of *Arabidopsis DR5::GUS* and *PIN1::GUS* seedlings non-exposed (Control, (**A**,**D**)) or exposed to 60 µM CdSO_4_ (Cd, (**B**,**E**)) or 400 µM Na_2_HAsO_4_·7H_2_O (As, (**C**,**F**)). Bars = 20 µm (**A**–**C**), 30 µm (**D**) and 50 µm (**E**–**F**).

It is also known that auxin biosynthesis and distribution in the root system is altered by heavy metals/metalloids, including Cd and As [17,22,35,36,37]. In fact, by monitoring the endogenous IAA distribution by the use of a *DR5::GUS* auxin-reporter line, it has been demonstrated that Cd increases the auxin signal in all three root types in *Arabidopsis* [16,38], as exemplified by the LRs in Figure 2A,B. In contrast, Cd downregulates the expression of the IAA efflux-carrier *PINFORMED1* (*AtPIN1*) gene [38] (Figure 2D,E). Differently from Cd, As alone, or combined with Cd, reduces or totally inhibits the DR5-signal in ARs and LRs (Figure 2A,C) [16], whereas it increases it in the PR [35]. Notably, As also reduces/inhibits *AtPIN1* gene expression in LRs (Figure 2D,F) and ARs [16].

**Figure 3:**
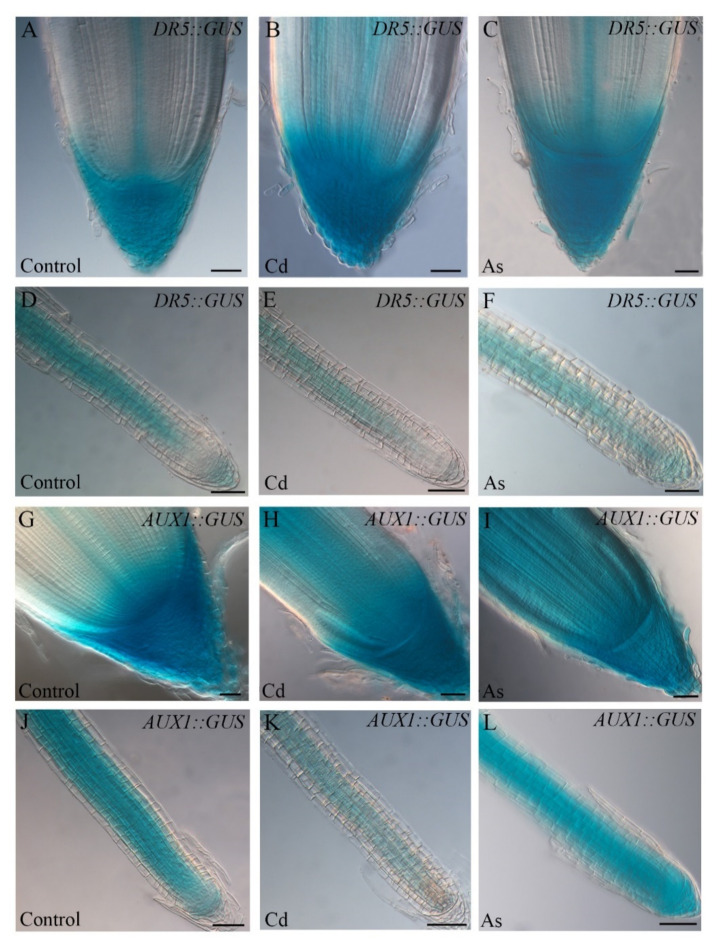
Expression patterns of *DR5::GUS* and *AUX1::GUS* in adventitious roots (ARs, (**A**–**C**,**G**–**I**)) and lateral roots (LRs, (**D**–**F**, **J**–**L**)) of *Oryza sativa DR5::GUS* and *AUX1::GUS* seedlings non-exposed (Control, (**A**,**D**,**G**,**J**)) or exposed to 100 µM CdSO_4_ (Cd, (**B**,**E**,**H**,**K**)) or 100 µM Na_2_HAsO_4_·7H_2_O (As, (**C**,**F**,**I**,**L**)). Bars = 40 µm.

Cadmium also modifies auxin homeostasis in rice by affecting the expression of specific auxin-related genes, which results in altered cell differentiation and root growth inhibition [17,39]. Histochemical analyses on a *OsDR5::GUS* line [40] has revealed that Cd reinforces and delocalizes the auxin signal in the apex of ARs, but not in the LRs [17,37] (Figure 3B,E), whereas As causes a diffused signal in the ARs, but not in the LRs [37] (Figure 3C,F). Thus, both pollutants disrupt auxin localization in the root meristems even if there are some differences related to the root type, i.e., ARs vs. LRs.

Anthranilate synthase, a heterocomplex consisting of ANTHRANILATE SYNTHASE ALPHA SUBUNIT 1/2 (ASA1/2) and BETA SUBUNIT 1 (ASB1), is a key rate-limiting enzyme of an early step of the tryptophan-dependent IAA biosynthesis [41,42,43]. Downstream of *ASA* genes, the *YUCCA* (*YUC*) gene family, encoding for flavinmono-oxygenase, converts the indole-3-piruvic acid into IAA [44].

In *Arabidopsis* hypocotyl, Cd increases the *INDOLE-3-PYRUVATE MONOOXYGENASE YUCCA6* (*YUC6)* gene expression and auxin levels. On the contrary, As, alone or combined with Cd, decreases *YUC6* expression and auxin levels, whereas the modification of auxin distribution in the LRs and ARs is coupled with a reduced expression of *PIN1* and *LAX3* after exposure to both Cd and As [16]. Furthermore, in the PR, Cd downregulates *PIN1*, which is considered the major non-redundant member of the family mediating the rootward auxin flow towards the QC [25,45].

Altogether these results indicate that the two toxic elements act on every component of the *Arabidopsis* root system by altering auxin biosynthesis, level, and transport [16].

In rice, the α-subunit of anthranilate synthase is encoded by *ANTHRANILATE SYNTHASE a1* and *a2* (*OsASA1* and *OsASA2*), whereas the *YUC* family of flavin monooxygenases includes at least fourteen genes ([46] and references therein). *OsASA2* is upregulated by abiotic stresses and its involvement in the plant response to Cd and As has been demonstrated [17,47]. *OsYUC1* has been also reported as a stress-related auxin gene [48,49]. This gene induces the expression of the transcription factor *WUSCHEL-related Homeobox* (*WOX*) gene, *WOX11*, which in turn drives AR and LR formation [50]. *OsASA2* and *OsYUC1* are differently affected by Cd and As, with the expression of *OsASA2* enhanced by As and not by Cd, and *OsYUC1* reduced by both pollutants [37]. As, instead, the expression of *OsYUC2* is enhanced by Cd, while not by As, it appears that different members of the YUC family can be differently sensitive to either pollutant [17].

Many aspects of rice root system development are under the control of auxin cellular transport both under normal and stressed conditions, as in *Arabidopsis*. For example, Cd affects the auxin-influx carrier *OsAUX1* expression in ARs and LRs in a different fashion, depending on the root type responding to Cd stress [51,52]. A strong *OsAUX1* expression is measured in the AR apex, while only low expression levels are detected in the LR apex (Figure 3H,K). In contrast, As reinforces *OsAUX1* expression in both AR and LR apices (Figure 3I,L) [37].

There are numerous members of the auxin efflux OsPIN family in rice [53]. As for *AtPIN1* expression in *Arabidopsis*, *OsPIN1* is expressed in the LRPs and is positively involved in the auxin-dependent AR emergence [54]. Furthermore, *OsPIN5b* is expressed in ARs and LRs, but As and Cd reduce/inhibit its expression in comparison to control treatments, with Cd having a stronger inhibitory effect on LRs than on ARs [17]. Altogether, the comparison of the effects of Cd and As on auxin homeostasis in ARs and LRs of rice and *Arabidopsis* underlines important similarities and differences between the two plant species.

## 3. Jasmonates and Ethylene Interact with Auxin in Changing the Root System under Cd and as Stress

Even if auxin is the core player in the control of rhizogenesis, stress hormones like jasmonates (JAs) and ethylene (ET) can regulate the division rate of the auxin-induced AR/LR initial cells. This suggests that stem cell activation may take part in developmental changes as a stress-avoidance-induced response involving a JA–ET crosstalk [55]. In accordance, a synergy between JA and auxin signaling pathways promotes root regeneration by activating root stem cells. Furthermore, a crosstalk between JA and ET signaling is critical for AR formation, at least in *Arabidopsis* [56]. Moreover, the involvement of JAs and ET and their crosstalk with auxin in the control of ARs and LRs in the presence of Cd and As pollution has been demonstrated.

Jasmonates include jasmonic acid methyl ester, i.e., methyl jasmonate (MeJA), JA, JA-isoleucine (JA-Ile) and the JA precursor 12-oxophytodienoic acid (OPDA), all involved in plant growth regulation and stress responses ([36] and references therein). Methyl jasmonate is the most active form of JAs. A positive role for JAs in auxin-induced LR and AR formation has been demonstrated in in vitro systems, e.g., in *Arabidopsis* ARs forming TCLs [56]. However, contrasting results have been obtained in planta, depending on different JAs concentrations and light conditions. In fact, from one side a negative role for JA in de-etiolation-induced AR formation has been demonstrated [57], whereas from the other side a positive one has been observed in dark-grown seedlings [56]. In addition, the application of MeJA to *Arabidopsis* dark-grown seedlings, or to TCLs growing in an IBA-containing medium, increases the expression of *ASA1*, confirming the interactive action of JAs and auxin in AR formation [12,42]. Moreover, in the same species, both early JA synthesis and signaling are involved in AR induction [56].

The upregulation of JA synthesis also promotes LR formation in *Arabidopsis* [58]. The JA receptor CORONATINE INSENSITIVE 1 (COI1) plays a critical role in the formation and distribution of LRs [59]. During LR formation, MeJA activates not only the transcription of *ASA1*, but also of several other auxin biosynthesis-related genes, such as *YUCCA2*, *YUCCA8* and *YUCCA9* and *ASB1*, and fails to increase LR initiation in mutants with disrupted auxin signaling [41,60,61]. Zhou and colleagues [55] report that the synergy between JA and auxin signaling pathways promotes rooting by activating the root stem cells by the induction of *ERF109*, *CYCLIN D6;1* (*CYCD6;1*) and *ERF115* expression and through a RETINOBLASTOMA-RELATED (RBR)-SCR-SHR network regulating asymmetric cell divisions, and activating the auxin-induced QC regulatory protein WOX5 in the QC and initial cells [10,55,62,63].

JAs also attenuate different abiotic stresses, including those caused by the exposure to heavy metals [64]. In *Arabidopsis*, Cd rapidly induces the expression of genes promoting endogenous JA synthesis, thus increasing the JA concentration in the roots. However, when formed or exogenously applied, JAs decrease the Cd concentration in root cell sap, by decreasing the expression of genes promoting Cd uptake and long-distance translocation, thereby attenuating Cd stress [65].

JAs are involved in mitigating the oxidative stress to which the plant, and in the first instance its root system, is exposed in the case of soil pollution, e.g., by reducing the detrimental effects of reactive oxygen species (ROS). When combined with Cd, MeJA reduces the oxidative stress in rice seedlings, improving the antioxidant response and lowering Cd accumulation. MeJA also reduces the As-induced lipid peroxidation of membranes [66,67]. Interestingly, the exposure to Cd and/or As does not change LR density in the rice *coleoptile photomorphogenesis2* (*cpm2*) mutant, which is blocked in the conversion of allene oxide to OPDA, but enhances the lipid peroxidation, evaluated as malondialdehyde levels, particularly in As presence [67]. Moreover, *OsASA2* and *OsYUCCA2* expression are affected by both pollutants and MeJA in the rice root system. However, As and Cd affect IAA and JAs levels in different ways. Interestingly, when combined with As or Cd, MeJA increases LRs in the wild type and reduces the length of the seminal ARs, suggesting that JAs might function as counteractors of As/Cd effects specifically on the LRs, at least as long as the pollutant toxicity is not too high [67].

The JA effects in mitigating the stress caused by soil pollution occur by a cascade of physiological/morphogenic responses also involving other hormones, like ET ([68] and references therein). Many ethylene responsive factors (ERFs) are common mediators of stress and developmental programs, including rooting [69]. In accordance with an ET role as a stress/developmental hormone, the ERF transcription factors are upregulated during AR formation and in response to wounding [70].

In the *Arabidopsis* PR, ET induces the *ASA1/WEAK ETHYLENE-INSENSITIVE2* (*ASA1/WEI2*) and *ASB1/WEI7* genes, and is involved in regulating the transcription of *PIN1*, *PIN2*, and *PIN4* IAA-efflux carriers, and *AUX1* influx-carrier [41,71,72]. Based on the analysis of *asa1/asa2* double mutant, as well as on GUS and in situ hybridization assays, it has been demonstrated that ET prevents AR formation in *Arabidopsis* by inhibiting both the anthranilate synthase genes and the *YUC6* gene. Moreover, it has been demonstrated that an IAA influx, triggered by AUX1 and LAX3, is necessary for ET action [73]. In addition, the effect of both Cd and As on every component of the *Arabidopsis* root system not only involves an alteration of auxin biosynthesis, level, and transport, but also an interaction with ET [16,28]. In rice, OsERF3 acts as a WOX11-interacting partner in AR development, suggesting the existence of an ET/auxin circuit in AR formation also in this species ([73,74] and references therein).

Ethylene and JA signaling are integrated with the auxin circuit in root development largely through transcription factors acting as key crosstalk nodes. The transcription factors EIN3 and its homologous EIL1 control most of the ET responses. The JASMONATE ZIM-DOMAIN (JAZ) proteins are the target of COI1 protein, and COI1–JAZ is a co-receptor of JA-Ile. Mutations affecting COI1 compromise the formation of the JA-Ile receptor complex, as occurs in *Arabidopsis coi1* mutants, which, as a consequence, are not only JA/MeJA-insensitive, but also insensitive to the ET-precursor 1-aminocyclopropane-1-carboxylic acid (ACC) [56,75]. Moreover, EIN3 and EIL1 physically interact with numerous JAZs, suppressing their activity [76]. In the presence of JA-Ile, JAZs are degraded releasing EIN3/EIL1, which still need ET for stabilization [77]. In accordance, the *ein3eil1* mutant is insensitive to both JA and ET, as confirmed in *Arabidopsis* TCLs during AR formation [56]. EIN2, a membrane protein of the endoplasmic reticulum, participates in ET signaling upstream of EIN3/EIL1 transcription factors, and downstream of the ET-receptor family [78]. At least in *Arabidopsis* TCLs, EIN2 acts as a functional link in the perception of JA and ET through a crosstalk with COI1 [79]. In addition, in rice, OsEIL1 has been demonstrated to directly activate the expression of *OsYUC8* to modulate auxin biosynthesis and ET-inhibited PR elongation. This further corroborates the hypothesis of a JA/ET/auxin interaction through EIN2, EIN3/EIL1, and COI1 in root growth control [80]. Interestingly, the above mentioned interaction is also involved in the competitive modulation of another developmental program, i.e., xylogenesis [56,81]. The ectopic formation of protoxylem and metaxylem (xylogenesis in planta) occurs in *Arabidopsis* starting from ACDs in the basal hypocotyl pericycle cells which divide periclinally instead of anticlinally, as it occurs in AR formation. The developmental process of xylogenesis is an auxin-induced developmental process, generally happening as a stress response [82] and it requires ET cooperation through the EIN3/EIL1 network [56,81]. Xylogenesis is also enhanced by increasing MeJA concentrations which repress AR formation [56]. However, the possibility that Cd and As can cause a reprogramming of ACDs from adventitious rooting to xylogenesis through an effect on the JA/ET crosstalk system affecting root system plasticity in *Arabidopsis* and/or rice still remains to be investigated.

ET, as Cd, is a positive regulator of ROS production, thus increasing per se the cellular oxidative stress [83,84]. In accordance, it has been shown that ET biosynthesis increases after Cd exposure and that the Cd-induced oxidative stress affects ET signaling, suggesting a crosstalk between the two pathways [85]. Recently, the promotion of ACC synthesis by Cd has been confirmed in *Arabidopsis*, and it has been proposed that the roots are the command center for ACC/ET to engage the proper Cd-stress response in the aerial organs [86]. Moreover, a crosstalk between JA and ET signaling is also active in response to Cd toxicity. In fact, in *Arabidopsis* Cd-exposed plants, the signaling pathways of both hormones are activated to mediate the stress-initiated nitrate allocation to roots to enhance Cd tolerance [87].

## 4. BRs and Their Crosstalk with JAs and ET in Root System Growth and Response to Pollution

Numerous studies suggest that brassinosteroids (BRs) play important roles during root growth and development. In fact, mutants impaired in BR biosynthesis or signal transduction display a short-root phenotype [88]. BRs are perceived by the plasma membrane-localized BRASSINOSTEROID INSENSITIVE1 (BRI1) and its two paralogs, BRI1-LIKE 1 (BRL1) and BRL3 ([89] and other references therein). BRI1 binds to brassinolide (BL), the most active form of BRs, and the ligand-mediated BRI1 receptor activation results in transphosphorylation events, which also involve co-receptors, like BRI1 ASSOCIATED KINASE1 (BAK1). Downstream of BRI1 and its co-receptors, the signal is transduced through several proteins until the final transcription factors BRI1-EMS SUPPRESSOR1 (BES1/BZR2) and BRASSINAZOLE-RESISTANT1 (BZR1) which ultimately regulate plant growth and development [90,91]. These transcription factors, in fact, can activate or repress the expression of hundreds of target genes in the *Arabidopsis* genome, mediating many aspects of plant development [92,93]. Moreover, BZR1/2 have been shown to directly regulate the AUXIN RESPONSE FACTORS (ARF) transcription factors, which are involved in realizing the transcriptional output of auxin ([94] and references therein). For example, in *Arabidopsis*, ARF6, that positively regulates AR formation in planta and in TCLs with ARF8 [56], directly interacts with BZR1 and this direct crosstalk is thought to integrate and specify BR and auxin signaling output [94]. In addition, an interaction between BR signaling and the PIN-LIKEs (PILS) proteins of auxin transport facilitators has been demonstrated, suggesting that BRs also affect auxin transport ([94] and references therein).

In rice, the reduced AR response of a mutant possessing a dysfunctional *BRASSINOSTEROID-6-OXIDASE* (*OsBR6OX*) gene involved in BR biosynthesis has helped to demonstrate that BRs are implicated in the initiation and development of crown (AR) roots [95,96]. Moreover, auxin treatments increase the expression of the rice BR receptor gene *OsBRI1*, whose promoter contains an auxin-response element (AuxRE) that is targeted by ARFs. In accordance, *OsBRI1* expression is downregulated in an *arf* mutant, suggesting that some ARFs control the degree of BR perception required for normal root development in rice [97]. The observation that auxins increase the expression of both the BR-receptor and of the BR-responsive genes also in *Arabidopsis*, strengthens the possibility that auxins control the degree of BR perception by regulating the expression of BR-related genes in a conserved way between the two plant species [98].

As previously mentioned, the QC is essential for the specification of the stem cell niche and for the maintenance of the undifferentiated state of stem cell initials in all root types of both rice and *Arabidopsis*. In addition to auxin, BRs are also required to control QC identity and stem cell activity, together with root meristem size, as shown in *Arabidopsis* PR [99,100]. In accordance, BL treatments can increase the expression of the QC marker *WOX5*, which is a gene active both in rice and *Arabidopsis* PR, LRs, and ARs, and whose expression is under auxin control [10,63,99,101]. BR signaling controls, at least partially, three separate functions in *Arabidopsis* PR development: cell division, cell elongation rate, and termination of cell elongation [102]. This is in accordance with studies on the leaf organ, where BR production and signaling differently modulate cell division and expansion [103].

BRs also regulate LR development through an interaction with auxin in a dose-dependent manner. In fact, BL has been shown to promote the initiation of LR primordia at low concentrations by increasing acropetal auxin transport, whereas at high concentrations BL suppresses LR formation at least in *Arabidopsis* [100,104,105]. It has been hypothesized that requirements for different BR levels occur in different root developmental zones, thus explaining both synergistic and antagonistic BRs and auxin interactions in different stages/aspects of root development [100,106]. However, even if a BR and auxin synergism seems to be prevalent at least for root induction, BR/auxin interdependency/cooperation is complex, because the two hormones regulate each other mutually on multiple levels [107].

In *Arabidopsis*, interactions between BRs and ET have been reported in the regulation of root elongation with BRI1 activity resulting in an enhanced expression of ET biosynthesis genes, followed by an accumulation of ACC and an enhanced ET signaling [108]. In addition, a cross-regulation between BRs and ET is known to be involved in controlling root growth and development [109]. A role for BRs in stress management in response to heavy-metal stress, including Cd stress, has also been demonstrated ([110] and references therein). Moreover, BRs increase salt tolerance in numerous plants, such as rice and *Arabidopsis* ([111] and references therein). In the latter, ET signaling facilitates salt stress-induced reassembly of microtubules (MTs), with an involvement of EIN3 [112]. BR signaling is also able to mediate salt tolerance by regulating ET biosynthesis and signaling [88,113]. Moreover, recent studies indicate that BR signaling also affects cytoskeleton functions including those of MTs and associated proteins, collectively suggesting that ET and BRs jointly affect MTs under multiple stress/developmental conditions ([114] and references therein).

It is known that a coordinated developmental and auxin-dependent remodeling of MTs is involved in the induction of ACD activity, leading to LR/AR initiation. This contributes to the shift from cell differentiation to cell division and vice versa [115]. Thus, an interaction between BRs and ET on MTs remodeling is possible in the initial cells of the root system. In accordance, BR signaling also directs formative cell divisions in *Arabidopsis* root meristems [116].

In addition, in *Arabidopsis* BRs regulate QC quiescence and the expression of the ET-induced *ERF115*, which is a limiting factor for QC divisions. These signals converge with JA signaling [117]; in fact, the JA-insensitive mutant *coi1-2* is responsive both to ERF115 and BR, showing an effect on QC division [55]. However, the integrated effect of BRs, ET, and JA in the initial events of the LR and AR rooting processes is still an open question.

In any case, as the exogenous BR treatments enhance not only ET levels, but also JA levels, a crosstalk among the three hormones is highly possible, at least in the signaling pathway inducing stress tolerance ([118] and other references therein). In accordance, BRs have been demonstrated to stimulate JA biosynthesis under stress in *Arabidopsis*, and to induce the expression of the JA biosynthetic gene *OXOPHYTODIENOATE-REDUCTASE 3* (*OPR3*) [119,120].

There are many studies on the effects of BRs in increasing plant tolerance to abiotic stresses, including heavy metals ([121,122] and references therein). BR treatments can reduce Cd accumulation and toxicity in numerous species, e.g., in *Brassica juncea* which belongs to the same family of *Arabidopsis thaliana* [123]. Moreover, the lipid peroxidation induced by Cd is reduced by BR supplementation and the negative effects of Cd are overcome by BR application through increased activities of antioxidant enzymes in numerous plants including *Arabidopsis* and rice [121,124].

In contrast, there is only a limited knowledge of the effects of BR application to plants exposed to As pollution. However, it has been proved that As stress causes both the activation of antioxidative enzymes, and of BR synthesis in *B. juncea* [125]. In addition, the application of 24-epibrassinolide (eBL) reduces As content in leaves of rice seedlings grown in hydroponic solution. Collectively, these data suggest that BRs could also limit the accumulation of As and increase the tolerance to the metalloid [126].

Very recently, it has been shown that the rice Glycogen Synthase Kinase3 (GSK3)-like kinase OsGSK2 integrates the JA and BR signaling pathways and triggers rice antiviral resistance [127]. This result not only provides a new insight into the crosstalk between JA and BR signaling, but also suggests that the same mechanism might also be active in abiotic stress responses.

## 5. Reactive Oxygen and Nitrogen Species Take Part in Root System Adaptations to Soil Pollution through an Interaction with Auxin/JA/BR and ET, Mediated by NO

As widely documented, ROS and reactive nitrogen species (RNS) are damaging by-products of the plant stress response, and the exposure to soil pollution enhances their formation. However, the regulation of root development is also controlled by ROS and RNS, in addition to auxin, JAs, ET, and BRs, independently from the stress exposure. In fact, endogenously generated ROS/RNS act as signaling molecules during PR and LR growth in both *Arabidopsis* and rice [26,37,128,129,130].

Root growth is profoundly affected by endogenously generated ROS, which are responsible for a balance between cell proliferation and differentiation ([109] and references therein). ROS interact with various hormones, particularly in plants exposed to heavy metal stress. Under Cd exposure, an increase of lipoxygenase activity, followed by lipid peroxidation, generally occurs, thus enhancing the production of oxylipins, including JAs. This event is crucial in rice because it reduces oxidative stress, thereby improving the antioxidant response and lowering Cd accumulation [67]. Furthermore, BRs regulate root growth interacting with ROS [109], and a parallel input of BR and JA in the QC quiescence and in the induction of *AP2/ERF115* is not only ET-responsive, but also ROS-responsive, as shown in *Arabidopsis* [55]. Collectively, present data show that the interaction between JA, ET, and BR involves ROS activity.

ROS and RNS metabolism occurs within peroxisomes, where the generation of hydrogen peroxide (H_2_O_2_) and nitric oxide (NO) occurs, as well as the production of H_2_O_2_ scavengers, including catalase (CAT) and ascorbate peroxidase (APX) [131]. One of the peroxisomal functions is, in fact, to contribute to cellular redox homeostasis by controlling the levels of ROS/RNS, either by producing or scavenging them, mainly activating the enzymatic/non-enzymatic antioxidant systems ([26] and references therein). Under normal conditions, peroxisomal ROS concentration is controlled; however, under Cd exposure, peroxisomal ROS homeostasis is disrupted and RNS are generated ([132,133] and references therein). Nitric oxide is a very important RNS and is also involved in many physiological processes in response to stress [134]. Nitric oxide is produced in numerous organelles, but also in the cytosol by the activity of nitrate reductase (NR) [135]. The peroxisomal NO seems to be produced by an L-arginine-dependent NO synthase (NOS)-like activity using NADPH as electron donor, as demonstrated in *Arabidopsis* [136]. Under stress conditions, the NO derived from various organelles, including the peroxisomes, accumulates in the cytosol [137].

The conversion of IBA into IAA also occurs in the peroxisomes through the β-oxidation pathway [138]. The peroxisome-originated auxin is important for LR and AR formation ([12,139] and other references therein), and NO is also generated during this conversion in *Arabidopsis* [12,140].

In plant cells, Cd and As exert their toxicity by inducing an imbalance between ROS and RNS production and detoxification. NO acts as a signaling molecule, coordinating development and stress responses, but it can also act as an oxidative stress inducer in *Arabidopsis* and rice, depending on its cellular concentration [26,37,130]. Arsenic is mainly present in two inorganic forms in the soil, arsenite and arsenate. Even if the two forms use different transport systems to enter the plant cell [141,142], arsenate is easily reduced to arsenite after cell entrance, and this reaction contributes to increasing the ROS levels in the cytosol [143].

By the use of transgenic *Arabidopsis* plants expressing a fluorescent protein fused to the PEROXISOMAL TARGETING SIGNAL 1 (PTS1), it has been recently demonstrated that Cd and As alter peroxisome distribution and size in *Arabidopsis* roots as well as peroxisomal NO production. The PR was specifically susceptible to peroxisome alteration, and Cd was more toxic than As under the tested conditions (i.e., plates vertically incubated at long-day conditions for 12 days on a full-strength Murashige and Skoog agarized medium containing 60 μM CdCl_2_ or 60 μM KH_2_AsO_4_) [26].

In a wide range of abiotic stresses, including Cd and As stresses, NO reacts with a wide range of proteins, but mainly with ROS, as observed both in *Arabidopsis* and rice [130,136,144]. Peroxynitrite is an example of RNS formed by the reaction between NO and the superoxide anion [145]. Its synthesis reduces NO levels in *Arabidopsis* [146]; in fact, an increase in peroxynitrite levels occurs in the roots of this plant exposed to Cd [136]. In rice ARs and LRs, Cd and As reduce NO levels, and NO decreases Cd and As uptake. NO also reduces the Cd-induced ROS levels by triggering peroxynitrite production, but this does not occur in the presence of As, highlighting a different response to Cd and As toxicity, which depends on the NO interaction with the specific pollutant at the tested concentration [130].

Numerous studies on heavy metals/metalloids tolerance have shown that ROS and NO can either cause an oxidative/nitrosative stress, or function as signaling molecules based on the reciprocal cell levels [130,147]. In Cd-exposed rice plants, an increased tolerance to the heavy metal occurs when the NO-donor sodium nitroprusside (SNP) is supplied [148]. Recently, it has been demonstrated that treatments with SNP enhance the NO-levels in rice ARs and LRs, which were reduced by Cd or As at the tested concentrations [130]. However, this NO enhancement resulted in an alleviation of the morphological and histological alterations induced by Cd, but not of those due to As, and in a different effect on the ROS/RNS balance depending on the pollutant [130].

**Figure 4:**
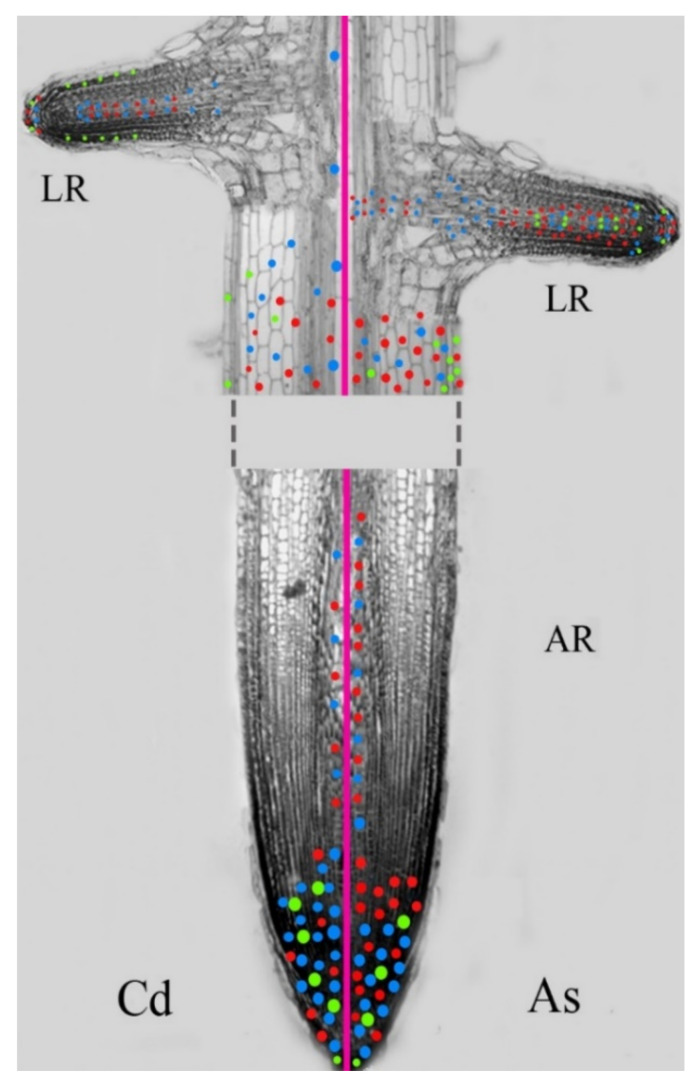
Model of indole-3-acetic acid (IAA) localization (blue dots), *AUX1* expression (red dots) and nitric oxide (NO) signal (bright green dots) in an adventitious root (AR) and in lateral roots (LRs) of rice seedlings grown for 10 days in the presence of Cd or As.

In several plants, NO interacts with auxin in various developmental processes including PR, LR, and AR formation, under both physiological and stress conditions including the heavy metal/metalloid reaction (Figure 4) [149]. In accordance, an increased NO production occurs after exogenous application of auxin and in auxin-overproducing mutants in numerous plants, including *Arabidopsis* [140] and rice [150]. However, by using SNP, it has been demonstrated that NO can also modulate the levels of auxin, by affecting its synthesis, transport, signaling, and degradation. In fact, *Arabidopsis* mutants with altered NO levels show changes in auxin biosynthetic enzyme activity, unbalancing auxin levels and changing the root meristem structure [151]. In rice seedlings, Cd and As alter auxin biosynthesis, levels, and distribution (see Section 2), but the SNP-derived NO counteracts the effects of both pollutants on auxin distribution and enhances *OsAUX1* expression, mainly in As presence [37]. In addition, treatments with exogenous auxins (IAA/IBA) increase the NO content only under Cd stress, while not under As, highlighting a Cd-specific mitigation effect of the exogenous auxins supplied, under specific concentration ranges [37]. Interestingly, in the same research it was shown that a buffering role for NO on the auxin distribution/influx alterations induced by Cd or As is present in both root types (ARs and LRs), suggesting that NO acts downstream of auxin and with the possible cooperation of other phytohormones [37].

All major classes of plant hormones may influence, at least to some degree, the endogenous levels of NO; on the other hand, NO may also affect biosynthesis, catabolism/conjugation, transport, perception, and/or transduction of different phytohormones, such as auxins, ET, JAs, and BRs [152]. It is also possible that NO derivates (e.g., peroxynitrite) interact with certain hormones, inducing the formation of products with altered biological activity [152]. Furthermore, NO controls per se a number of cytoskeleton-mediated processes in plants, such as root growth and development; also, a NO-guided rearrangement of MTs is accompanied in *Arabidopsis* by the acceleration of PR growth ([153] and other references therein). This occurs in particular in the PR elongation zone and affects the epidermal cells, as shown by the application of a set of NO-modulating chemicals such as exogenous NO donors (SNP) or NO-scavangers (2-(4-carboxyphenyl)-4,4,5,5-tetramethylimidazoline-1-oxyl-3-oxide, c-PTIO) [153].

In the pericycle cells of *Arabidopsis* basal hypocotyl, NO is detected at the early stages of both AR formation and xylogenesis, and its production is enhanced by MeJA [154]. Interestingly, the IBA/IAA-induced adventitious rooting is increased by MeJA to a similar extent as xylogenesis, suggesting a role for JA in modulating adventitious rooting and xylogenesis programs in the same target cells through an interaction with NO, whose signal is enhanced early by MeJA [154]. Since ARs are induced in the *Arabidopsis* hypocotyl by auxin (through the induction of anticlinal ACDs) in competition with xylogenesis (occurring via periclinal ACDs) [32], it is possible that a NO-guided rearrangement of the cytoskeleton and of cell plate orientation occurs, thereby changing the developmental program. In fact, the cell plate formation is known to be sensitive to changes in the microtubular cytoskeleton, and to be affected by nitrotyrosine, a product derived from NO-mediated post-translational modification ([155] and references therein). In addition, in *Arabidopsis*, increased NO levels induce changes in the actin cytoskeleton, where the synthetic auxin 2,4-dichlorophenoxyacetic acid affects cytoskeleton and peroxisomal dynamics by a promotion of *S*-nitrosylation and oxidation of actin [156].

Interestingly, the AR primordia of *Arabidopsis* do not need prolonged NO activity, perhaps because they are capable of sustaining their growth after the QC is established, which occurs at stage VII of development [10], i.e., exactly when the NO signal is quenched [154]. In accordance, it has been suggested that when NO production is auxin-dependent it may occur exclusively under specific temporal and spatial contexts [152].

In some developmental processes, a crosstalk between NO and JA has been reported as probably mediated by *OPR3*. In *Arabidopsis*, OPR3, which is involved in JA biosynthesis, is located in the peroxisomes where its expression is increased by NO, resulting in an increased JA production ([157] and references therein). As also auxins induce NO formation, as previously described, a cooperation between auxin and JA through the NO node is highly possible.

An antagonistic relationship between NO and ET is known in numerous processes. However, some reports have indicated that NO donors, such as SNP, sometimes, and mainly under stress conditions, stimulate ET production, rather than repress it [152]. For example, increases in both ET and NO have been observed in *Arabidopsis* roots subjected to Fe deficiency [158]. Moreover, using *Arabidopsis* NO-deficient mutants characterized by early senescence, mutations in *EIN2* suppress this phenotype, suggesting that the EIN2 protein might play a key role in the crosstalk between ET and NO signaling pathways [159].

Brassinosteroids have been reported to utilize H_2_O_2_- and NO-mediated mechanisms to provide stress tolerance, as demonstrated in *Arabidopsis* [160]. In this species, NO production mediates the BR-triggered modifications in root architecture [161]. Other studies have shown that NO, ROS, and mitogen-activated protein kinase (MAPK) cascade are associated with BR responses followed by the development of resistance, e.g., to cold [111]. In addition, BRs induce the production of endogenous NO by stimulating the activity of NOS-like and NR enzymes and, at the same time, promote the development of ARs, at least in cucumber [162]. However, the link between BRs and NO in root formation is still poorly understood because the interaction mechanisms and signal transduction pathways of BRs and NO still need to be investigated by molecular and genetic methods [162]. In this regard, the use of the model plants *Arabidopsis* and rice will provide promising data to unravel novel root growth regulation mechanisms.

Taken together, the scenario described here highlights an important role for the signaling network mediated by NO in the root responses to soil pollution due to Cd and/or As, involving the interaction of this gaseous molecule with auxins, JAs, BRs, and ET.

## 6. Conclusions and Perspectives

The results summarized in this review are focused on the poorly known role of the crosstalk between JA/BR/ET on the cellular events leading to AR and LR formation in the monocot rice and in the dicot *Arabidopsis* in the presence/absence of the soil pollutants Cd and As.

In developmental biology, the knowledge of the mechanisms underlying BR-/JA-/ET-regulated AR and LR formation, mediated by NO signaling, helps us to understand the trade-off between growth and adaptation in the presence of a stress due to Cd/As. For applicative purposes, insights into the same mechanisms provide useful tools for the optimization of rice cultivation in heavy metal-/metalloid-polluted soils, and for strategizing new approaches to obtain rice root systems with efficient abilities to sustain the crop biomass in presence of the pollutants.

## Data Availability

Data sharing not applicable.
